# Heart Rate Estimation Using the Galaxy Watch During Maximal Cardiopulmonary Exercise Testing: Cross-Sectional Validation Study

**DOI:** 10.2196/81917

**Published:** 2026-04-16

**Authors:** Allan Inoue, João Paulo Ferreira Soares, Felipe Antunes-Santos, Alexandre Ferreira, Alberto Gonçalves, João Arthur Alcântara, Marcelo Rodrigues dos Santos

**Affiliations:** 1 Health Data Lab, Sidia Institute of Science and Technology, Av. Autaz Mirim, 2211 - Distrito Industrial I, Manaus, 69075-155, Brazil, 55 (92) 3212-3444

**Keywords:** photoplethysmography, smartwatches, wearable, maximal exercise, cardiopulmonary exercise test

## Abstract

**Background:**

Photoplethysmography-based smartwatches are increasingly used for continuous heart rate (HR) monitoring. Their accuracy has been demonstrated at rest or during low-intensity activity, but data are scarce for maximal-intensity exercise, when motion artifacts and rapid hemodynamic changes can degrade the photoplethysmography signal. Validating these devices under such demanding conditions is essential before they are applied to clinical exercise testing, athletic training, or remote health monitoring.

**Objective:**

This study aimed to evaluate the validity of the Samsung Galaxy Watch 6 (GW6) in estimating HR throughout a graded, maximal ramp cardiopulmonary exercise test performed on a treadmill. A secondary aim was to explore whether measurement error varies across 5 predefined intensity zones (50%‐60%, 60%‐70%, 70%‐80%, 80%‐90%, and 90%‐100% of the maximum HR determined individually for each participant).

**Methods:**

Overall, 55 healthy adults (30 men, 25 women; mean age 30.3, SD 8.2 years) completed a symptom-limited incremental treadmill protocol to volitional exhaustion. Simultaneous HR recordings were obtained from the GW6 (left arm) and a Polar H10 chest strap monitor, which served as the reference standards. For each intensity zone, the following agreement indices were computed: intraclass correlation coefficient (ICC), median absolute error, median absolute percentage error, and root mean squared error. Bland-Altman analysis was performed to quantify the mean bias and 95% limits of agreement between the GW6 and the Polar H10. Statistical significance was set at *P*<.05.

**Results:**

Agreement between the GW6 and Polar H10 varied across exercise intensities. ICC indicated moderate to good agreement at low to moderate intensities (ICC=0.71 at 50%‐60%; ICC=0.89 at 60%‐70%; ICC=0.54 at 70%‐80%; and ICC=0.64 at 80%‐90% HRmax), and at 90%‐100% of HRmax the agreement was good-to-excellent (ICC=0.90). Absolute error metrics showed stable or reduced errors with increasing intensity, with median absolute error consistently around 1‐3 bpm and median absolute percentage error declining from 2.90% at 50%‐60% HRmax to 0.60%‐0.75% at ≥70% HRmax. Root mean squared error ranged from 4.62 to 4.88 bpm across intensity zones. Bland-Altman analysis showed that the GW6 consistently underestimated HR compared with the Polar H10, with an overall mean bias of −2.67 bpm and wide limits of agreement (−16.90 to 11.57 bpm). This negative bias was present across all HR zones. The agreement was adequate for group-level comparisons but displayed substantial individual variability.

**Conclusions:**

The GW6 provides a good degree of validity for HR monitoring during a maximal treadmill cardiopulmonary exercise test in healthy young adults. Although measurement error increases modestly at near-maximal workloads, absolute errors remain well within clinically acceptable thresholds. These findings support the potential use of GW6 as a convenient, noninvasive alternative for HR tracking in laboratory-based exercise testing.

## Introduction

The use of wrist-worn wearables (eg, smartwatches) has been growing rapidly over the last years. These devices have become popular for monitoring various health parameters, including heart rate (HR) through photoplethysmography sensors. HR is estimated from photoplethysmography signals through the changes in blood volume beneath the skin [1]. Although convenient and noninvasive, photoplethysmography-based HR monitoring remains vulnerable to motion artifacts, sensor-skin interface issues, and light interference, particularly during dynamic or high-intensity exercise. Ensuring the validity of HR data under such demanding conditions is therefore critical, especially when wearables are used for exercise prescription or physiological assessment [2].

Several studies have evaluated the accuracy of wrist-based devices at rest or during light-to-moderate exercise, showing acceptable validity compared to electrocardiogram (ECG) or chest-strap references [3]. Thomson et al [3] compared HR values from the Apple Watch and Fitbit Charge HR2 with ECG during the Bruce Protocol across multiple intensity domains, from very light to very vigorous exercise. The Apple Watch showed lower relative error rates (2.4%‐5.1%) than the Fitbit (3.9%‐13.5%) across all intensities, with very high concordance (concordance correlation coefficient >0.90) at light workloads. However, HR accuracy progressively declined at higher intensities, indicating that optical sensors may lose precision during vigorous or near-maximal exertion. Importantly, that study did not include a maximal cardiopulmonary exercise test (CPET) or direct measurement of oxygen consumption.

To date, only 1 study [4] (conducted with the Apple Watch during a maximal CPET using a ramp protocol and simultaneous gas exchange analysis) has validated HR estimation under maximal physiological stress. The authors reported good to very good correlations with criterion HRmax and trivial mean bias compared with a Polar chest strap, although moderate interdevice variability was observed. These findings demonstrate that wrist-worn optical sensors can validly estimate HRmax during maximal effort, but evidence remains limited to a single device and a small sample size.

Given the lack of studies assessing the accuracy of smartwatches during maximal effort protocols, particularly those incorporating direct measurement of oxygen consumption, further validation is warranted. To date, no study has systematically evaluated the HR performance of the Samsung Galaxy Watch 6 (GW6) in young healthy individuals during a ramp incremental test with concurrent oxygen uptake analysis. By integrating continuous gas-exchange assessment with a standardized graded treadmill protocol, this study provides the first validation of the GW6 under conditions that reflect both physiological demand and methodological rigor; the only available investigation involved patients with coronary artery disease [5]. Accurate HR monitoring during CPET is fundamental for determining metabolic thresholds, exercise intensity zones, and overall cardiorespiratory fitness.

Therefore, this study aimed to determine the validity of the Samsung GW6 in estimating HR during a maximal ramp CPET performed on a treadmill in healthy adults, using the Polar H10 chest strap as the criterion measure. We hypothesized that HR values obtained from the GW6 would demonstrate at least moderate agreement (intraclass correlation coefficient [ICC] ≥0.60) with the reference device across 5 intensity zones (Z1–Z5) of the maximal ramp protocol.

## Methods

### Study Design

This is a cross-sectional study, within‐subject design to compare the HR accuracy from the GW6 with criterion measures of HR (Polar H10 chest strap) during a maximal ramp CPET conducted on a treadmill. All participants had their health conditions evaluated by a physician before the tests. Participants were instructed to abstain from alcoholic beverages and any medication for 24 hours, to avoid caffeine intake for 12 hours before the test, and to hydrate with water only. Body weight and height were measured using a weighing scale and stadiometer (InBody 770 analyzer), and skin type was measured via the Fitzpatrick skin type scale [6]. The environment in the laboratory was standardized throughout all sessions by maintaining an ambient temperature of approximately 22°C. All technical procedures followed the American College of Sports Medicine guidelines [7]. We followed the STROBE (Strengthening the Reporting of Observational Studies in Epidemiology) statement: guidelines for reporting observational studies [8].

### Participants

The inclusion criteria were healthy, recreationally active participants who engaged in at least 150 minutes of moderate-intensity activities and/or 75 minutes of vigorous activities per week.

Exclusion criteria were overweight and obesity, recent musculoskeletal injuries, any cardiovascular disease, and use of medications or supplements (eg, beta-blockers and caffeine) that impact HR measures. Participants signed the informed consent form after a verbal and written explanation of the activities to be carried out and a full understanding of the possible risks involved in participating in the study.

Initially, 76 participants began the CPET protocol. Four were excluded because of execution failures, and 17 were excluded because of device failures. The participant selection flowchart can be found in the Results section. Execution failure refers to problems that occurred before the trial started. Examples include the device not being set up correctly; failure to pair or synchronize the device; and incorrect entry of participant information into the device. Device failure refers to problems that arose during or after the trial. Examples include: loss of connection during CPET execution; the Polar H10 chest strap producing an unstable, missing, or abruptly dropping HR signal for any portion of the test; and devices that were correctly configured but still failed to generate all required output files.

### Maximal Ramp Cardiopulmonary Exercise Test

All participants performed maximal ramp CPET on a motorized treadmill (COSMED T170 DE) to determine HR at increasing intensities and maximal oxygen consumption (determined as the highest value observed during the test). The test was started at 5 km∙h^-1 and the speed increased by 1 km∙h^-1 every minute, with the slope kept constant at 1% [9] until they met at least two out of the following three conditions: (1) reached 90% of their maximal HR value calculated using Tanaka’s equation [10], (2) achieved a respiratory exchange ratio (RER) greater than or equal to 1.05 [11], and (3) reached maximal voluntary exhaustion, evaluated by the Borg (6-20) subjective scale of effort [12]. Gas exchange recording was performed using the K5 portable metabolic analyzer (COSMED). Device setup, calibration, and data extraction were conducted via COSMED OMNIA Metabolic software. The equipment was calibrated before each test as recommended by the manufacturer.

The test was terminated according to the termination criteria recommended by the American College of Sports Medicine guidelines [13], which include the occurrence of cardiovascular, respiratory, or musculoskeletal symptoms, abnormal blood pressure responses, significant rhythm disturbances, or voluntary exhaustion. All tests were supervised by a physician trained in CPET.

During the test, participants wore a chest strap HR monitor (Polar H10) and the GW6 smartwatch. HR data were evaluated across 5 intensity zones commonly used in various applications (50%‐60%, 60%‐70%, 70%‐80%, 80%‐90%, and 90%‐100% of HRmax). The average HR was calculated for each of the 5 intensity zones based on the maximal HR measured by the Polar H10 from the whole test. This approach is consistent with standard clinical practice in cardiology and incremental exercise testing and was chosen to provide a stable and representative estimate of the physiological response at each workload while minimizing the influence of beat-to-beat variability and transient signal artifacts.

Resting HR was measured using the Polar device before the start of the V̇O_2_ max test protocol. Participants were seated quietly for several minutes to ensure a stable baseline prior to data collection.

### Criterion Measure

The Polar H10 chest strap (Polar Electro Oy) served as the criterion method for measuring HR. This device has previously been validated against ECG [14]. Each participant was fitted with the Polar chest strap, set to collect continuous HR data throughout the protocol. On completion of the activity, data were transferred from each Polar transmitter to a central laptop to be viewed and analyzed using Polar Flow (version 6.25.2, Polar Electro Oy).

### Wrist-Worn Wearable Device

The GW6 (Samsung Electronics Co Ltd) is a multisensor smartwatch. Accelerometer, gyroscope, barometer, magnetometer, and photoplethysmography are some of the GW6’s sensors. During our experiments, all GW6s were connected to separate smartphones (Galaxy A22, Samsung Electronics Co Ltd), and all HR data were exported using the Samsung Health app. In cases where the activity data needed to be trimmed, full health data were exported via the Samsung Health app, with the raw data CSV file processed using Python (version 3.12.7; Python Software Foundation).

Participant height, date of birth, and wrist orientation were entered into each device before the protocol. The GW6 was positioned on the left arm located just above the wrist, as per manufacturer instructions (2 cm above styloid process of radius). In addition, the GW6 was set to record the activity protocol as a workout, being started and stopped at the commencement and completion of the activity. The GW6 was set to record “treadmill run,” as this was the most relevant option available on the watches.

### Statistical Analysis

Sample size calculation was performed on a web-based calculator according to Arifin [15]. The lowest acceptable ICC was 0.50, and the expected reliability was ICC=0.75 [16]. The number of raters or repetitions considered was 2 (Polar H10 vs GW6). A significance level of *α*=.05 (2-tailed) and a power of 80% were set. As a result, 45 participants were needed for this study. However, considering a dropout rate of ~15%, we recruited 55 healthy adults, composed of 30 men and 25 women, aged between 18 and 59 years.

All data were analyzed using a custom-made Microsoft Excel spreadsheet [17]. Data are shown as mean (SD) or median (IQR). The validity of the wearable was determined by the following statistical tests: (1) Correlation between the wearable and the criterion measurement: ICC, common cut-off points for validity assessment: poor (<0.50), moderate (0.50 to 0.75), good (0.75 to 0.90) and excellent (>0.90) [16]; (2) Pearson correlation coefficient; (3) Systematic differences between the wearable and the criterion measurement: median absolute error (MAE) and median absolute percentage error (MAPE) compared to the criterion measurement (criterion-wearable)/criterion × 100). The MAPE was considered with acceptable error when <10% [18]. Additionally, root mean square error (RMSE) was calculated. A predefined subgroup analysis was conducted by stratifying participants according to skin tone using the Fitzpatrick classification. To preserve statistical power, Fitzpatrick categories were aggregated into 3 groups based on pigmentation level. Device performance was then evaluated within each group using the same accuracy and agreement metrics across exercise intensity zones. This subanalysis aimed to assess whether skin tone influenced HR estimation during the incremental cardiopulmonary exercise test.

Bland-Altman analysis was performed to quantify the mean bias and 95% limits of agreement (LoA) between the GW6 and the Polar H10 across the full dataset and within each predefined HR zone (50%‐60%, 60%‐70%, 70%‐80%, 80%‐90%, and 90%‐100% HRmax). For each comparison, the mean difference (device − reference), the corresponding SD, and the upper and lower LoA (mean difference, 1.96 SD) were calculated. Visual inspection was used to assess whether the differences were homoscedastic and whether any proportional bias was present across the range of HR values.

To further characterize agreement between the GW6 and the Polar H10 reference, we computed the coefficient of variation (CV%) for each intensity zone as CV%=(SD/mean) × 100, applying the formula separately to the GW6 and H10 HR values. Additionally, 2-way repeated measures ANOVA was performed to examine whether HR values obtained from GW6 differed from those recorded with the Polar H10 across the 5 predefined intensity zones (% HRmax).

### Ethical Considerations

The study protocol was approved by the Investiga Instituto de Pesquisa Ethics Committee (CAAE 62031722.4.0000.5599), and all procedures were conducted following the standards set by the Declaration of Helsinki. Data were anonymized before analysis to ensure confidentiality. No monetary or other forms of compensation were provided. The manuscript contains no identifiable information or images of individual participants. All participants were informed about the study procedures and provided written informed consent.

## Results

**Figure 1.**
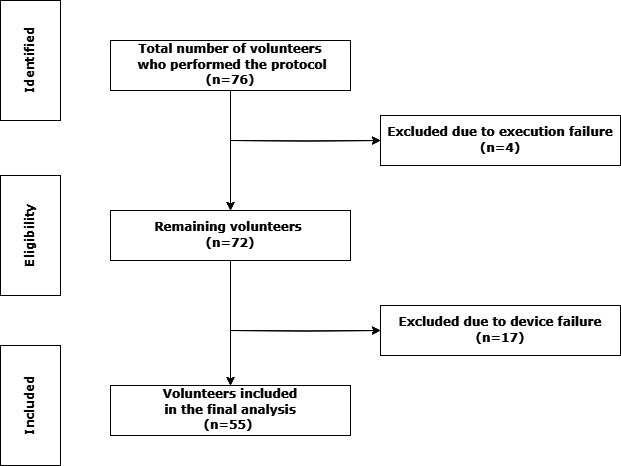
STROBE (Strengthening the Reporting of Observational Studies in Epidemiology) flow diagram.

Participant characteristics are presented in Table 1 and the flow diagram of participants is presented in Figure 1. The incremental CPET was stopped only after participants satisfied at least 2 of 3 predefined maximal-effort criteria: a Borg rating of perceived exertion ≥18, indicating volitional exhaustion; a HR reaching ≥90% of the age-predicted maximal HR calculated with Tanaka’s equation; and RER ≥1.05, reflecting near-maximal metabolic stress. Of the 55 participants, all 55 (100%) met the HR criterion, 31 (56%) achieved a Borg score ≥18, and 40 (73%) attained an RER ≥1.05 (mean RER at termination 1.08, SD 0.07). When the criteria were examined in combination, 31 participants fulfilled both the Borg and HR conditions, 16 participants met both the Borg and RER conditions, and 40 participants satisfied the HR and RER conditions; among these, 16 participants fulfilled all 3 criteria simultaneously.

**Table 1.** Participant characteristics.

Variables	Male (n=30)	Female (n=25)	All (n=55)
Age (years), mean (SD)	29.8 (8.6)	31 (7.7)	30.3 (8.2)
BMI (kg/m^2^), mean (SD)	71.7 (8.1)	60 (9.6)	66.3 (10.6)
Height (cm), mean (SD)	170 (6.1)	158.5 (4.7)	164.8 (8)
Fitzpatrick skin type, n (%)			
II	0 (0)	1 (1.8)	1 (1.8)
III	4 (7.3)	10 (18.2)	14 (25.5)
IV	16 (29.1)	12 (21.8)	28 (50.9)
V	8 (14.5)	2 (3.6)	10 (18.2)
VI	2 (3.6)	0 (0)	2 (3.6)
Resting HR^a^ (bpm^b^), mean (SD)	63.1 (8.7)	70.8 (9.7)	66.6 (9.9)
RER^c^max^d^, mean (SD)	1.09 (0.04)	1.06 (0.08)	1.08 (0.07)
V̇O₂max^e^ (mL∙kg∙min-1), mean (SD)	46.8 (5.5)	41.4 (7.7)	44.3 (7.1)

a HR: heart rate.

b bpm: beats per minute.

c RER: respiratory exchange ratio.

d max: maximum.

e V̇O₂max: maximal oxygen consumption.

Descriptive values, correlations (Pearson and ICC), MAE, MAPE, and RMSE are presented in Table 2. At low intensities (50%‐60% HRmax), agreement was moderate (ICC=0.71; 95% CI 0.55-0.82), with a MAE of 3.0 bpm and a MAPE of 2.9%. Agreement improved at moderate intensities (60%‐70% HRmax), reaching good levels (ICC=0.89; 95% CI 0.82-0.93), accompanied by a reduction in relative error (MAPE=1.77%). At vigorous intensities, ICC values declined, indicating reduced between-subject agreement (ICC 0.54; 95% CI 0.32-0.70 at 70%‐80% HRmax and ICC=0.64; 95% CI 0.45-0.77 at 80%‐90% HRmax). Despite this reduction, absolute and relative error metrics improved substantially, with MAE approximately 1.0 bpm and MAPE below 1%, suggesting higher point-wise accuracy. At maximal intensities (90%‐100% HRmax), agreement increased markedly, reaching good-to-excellent levels (ICC=0.90; 95% CI 0.83-0.94), while error metrics remained low (MAE=1.0 bpm; MAPE=0.60%). RMSE values were relatively consistent across intensity zones, ranging from 4.37 to 4.88 bpm.

**Table 2.** Galaxy Watch 6 heart rate and comparisons with criterion (Polar H10). Median absolute error and median absolute percentage error are reported as median (IQR). In the FITZ column: Groups II-III: 15 participants; IV: 28 participants; V-VI: 12 participants; all: 55 participants.

%HRmax^a^ and FITZ^b^		H10^c^ (BPM^d^), mean (SD)	GW6^e^ (BPM)	ICC^f^ (CI 95%)	Pearson *r*^g^ (CI 95%)	Median AE^h^ (IQR)	Median APE^i^ (IQR)	RMSE^j^
50-60								
	II-III	99.87 (6.01)	95.40 (4.82)	0.73 (0.36-0.90)	0.73 (0.35-0.90)	3 (2-4)	3.16 (2.06-3.93)	4.57
	IV	99.04 (4.92)	95 (5.52)	0.62 (0.33-0.81)	0.62 (0.32-0.81)	3 (2-4)	2.97 (1.98-4.07)	5.23
	V-VI	97.17 (5.57)	95.42 (6.11)	0.93 (0.77-0.98)	0.92 (0.74-0.98)	1 (0-1)	1.02 (0-1.06)	2.26
	All	98.85 (5.36)	95.20 (5.38)	0.71 (0.55-0.82)	0.69 (0.53-0.81)	3 (1-4)	2.90 (1.04-3.99)	4.62
60-70								
	II-III	118.07 (5.95)	113.53 (5.96)	0.68 (0.27-0.88)	0.66 (0.22-0.88)	3.50 (1.25-5)	3.10 (1.08-4.21)	4.64
	IV	116.89 (5.12)	113.25 (5.89)	0.58 (0.27-0.78)	0.58 (0.26-0.78)	2 (1-5)	1.79 (0.86-4.26)	4.99
	V-VI	115.67 (5.16)	114.42 (5.95)	0.97 (0.90-0.99)	0.97 (0.91-0.99)	1 (0-1.50)	0.83 (0-1.34)	1.35
	All	116.95 (5.33)	113.58 (5.83)	0.89 (0.82-0.93)	0.90 (0.83-0.94)	2 (1-5)	1.77 (0.85-4.05)	4.37
70-80								
	II-III	136.27 (7.24)	132.87 (6.82)	0.81 (0.52-0.93)	0.80 (0.48-0.93)	1.50 (1-2.75)	1.06 (0.73-2.01)	3.28
	IV	135.61 (5.78)	134.45 (10.46)	0.42 (0.06-0.68)	0.49 (0.14-0.73)	1 (1-2.75)	0.75 (0.74-2.03)	6.05
	V-VI	133.83 (6.75)	133.67 (6.87)	0.99 (0.96-1)	0.99 (0.96-1)	0 (0-1)	0 (0-0.75)	0.67
	All	135.40 (6.35)	133.85 (8.77)	0.54 (0.32-0.70)	0.70 (0.53-0.81)	1 (1-2)	0.75 (0.69-1.52)	3.96
80-90								
	II-III	155 (7.73)	151.87 (7.55)	0.46 (–0.04 to 0.78)	0.45 (–0.08 to 0.78)	1 (0-3)	0.66 (0-1.82)	2.83
	IV	154.50 (6.79)	153.21 (11.92)	0.39 (0.02-0.66)	0.44 (0.08-0.70)	1 (0-2)	0.64 (0.00 to 1.30)	7.71
	V-VI	151.58 (7.74)	150.92 (7.74)	0.96 (0.88-0.99)	0.96 (0.86-0.99)	0 (0-1)	0 (0-0.68)	1.17
	All	154 (7.24)	152.35 (9.95)	0.64 (0.45-0.77)	0.75 (0.60-0.84)	1 (0-2)	0.64 (0-1.34)	4.88
90-100								
	II-III	174.67 (8.81)	169.53 (10.93)	0.53 (0.05-0.81)	0.53 (0.02-0.82)	1 (0.25-3)	0.61 (0.14-1.62)	6.23
	IV	173.61 (7.75)	170.89 (8.97)	0.77 (0.57-0.89)	0.78 (0.57-0.89)	1 (0-3.50)	0.58 (0-2.01)	3.23
	V-VI	170.67 (6.97)	169.17 (5.57)	0.79 (0.41-0.93)	0.79 (0.39-0.94)	1 (1-1)	0.60 (0.58-0.62)	1.38
	All	173.25 (7.88)	170.15 (8.84)	0.90 (0.83-0.94)	0.87 (0.79-0.92)	1 (0.75-3)	0.60 (0.41-1.62)	4.52

a %HRmax: percentage of maximum heart rate.

b FITZ: Fitzpatrick Skin group.

c H10: Polar H10.

d BPM: beats per minute.

e GW6: Galaxy Watch 6.

f ICC: intraclass correlation coefficient.

g Pearson *r*: Pearson correlation coefficient.

h AE: absolute error.

i APE: absolute percentage error.

j RMSE: root mean square error.

**Figure 2.**
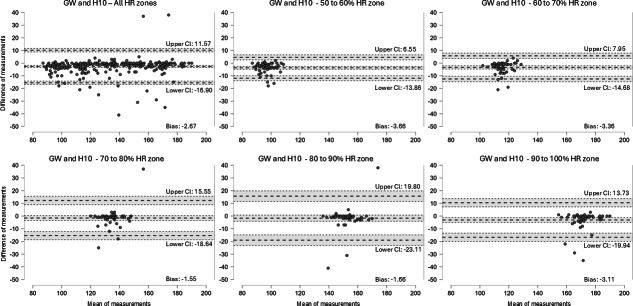
Bland-Altman analysis. GW: Galaxy Watch; HR: heart rate; H10: Polar H10 chest strap.

Across all HR measurements, the GW6 showed a systematic underestimation of HR relative to the Polar H10, with a mean bias of −2.67 bpm and 95% LoA ranging from −16.90 to 11.57 bpm. Although most points fell within these limits, the negative bias indicates that the smartwatch consistently reported lower values than the reference. When examined by HR zones, the pattern of underestimation persisted with small variations in magnitude: 50%‐60% HRmax: bias −3.66 bpm, LoA −13.86 to 6.55 bpm; 60%‐70% HRmax: bias −3.36 bpm, LoA −14.68 to 7.95 bpm; 70%‐80% HRmax: bias −1.55 bpm, LoA −18.64 to 15.55 bpm; 80%‐90% HRmax: bias −1.66 bpm, LoA −23.11 to 19.80 bpm; and 90%‐100% HRmax: bias −3.11 bpm, LoA −19.94 to 13.73 bpm (Figure 2). Although agreement was acceptable for group-level analysis, the width of the LoA demonstrates meaningful variability at the individual level, particularly at higher intensities where physiological oscillations are greater.

Additionally, the CV ranged between 4.6% to 5.4% for Polar and 5.1% to 6.5% for GW. ANOVA (device) revealed a significant main effect of Device (*P*<.001), indicating that the GW6 records a modestly lower HR than the Polar H10. The main effect of % HR was also significant (*P*<.001), reflecting the expected increase in HR across intensity zones. Importantly, the interaction (device vs % HR) was not significant (*P*=.73), demonstrating that the systematic underestimation is consistent across all % HRmax levels.

## Discussion

### Principal Findings

The primary aim of this study was to evaluate whether the GW6 provides HR estimates that are in at least moderate agreement with a criterion reference chest-strap (Polar H10) across 5 intensity zones (Z1-Z5) of a maximal ramp CPET. The data partially support our hypothesis. Moderate agreement was observed at lower intensities (50%‐60% HRmax), improving to good agreement at moderate intensities (60%‐70% HRmax). At vigorous intensities (70%‐80% and 80%‐90% HRmax), ICC values declined, before increasing again at maximal effort (90%‐100% HRmax), where agreement reached good-to-excellent levels. These findings indicate that between-subject agreement is not monotonic across exercise intensity and is influenced by changes in HR variability across zones. Importantly, despite fluctuations in ICC, absolute and relative error metrics demonstrated a consistent improvement with increasing intensity. MAE remained low across all zones (approximately 1‐3 bpm), while MAPE decreased progressively from 2.9% at low intensity to approximately 0.6% at vigorous-to-maximal intensities. These values are well within thresholds generally considered clinically acceptable for HR monitoring during exercise.

A “U-shaped” pattern in accuracy was observed, characterized by a decline in reliability (as measured by ICC) at intermediate workloads (70%‐80%), followed by a partial recovery at near-maximal intensities. This pattern reflects changes in the consistency of participant rankings (ICC) rather than absolute accuracy. While the ICC suggests reduced consistency in participant rankings at moderate to vigorous intensities, the absolute error metrics (MAE and MAPE) demonstrate a linear improvement in accuracy as intensity increased. The decline in reliability at intermediate workloads is likely attributed to transient mechanical artifacts, such as increased variability and amplitude of arm motion, which may interfere with optical signal stability. Conversely, near-maximal effort, arm movement often becomes more rhythmically constrained, which may help stabilize the photoplethysmography signal and partially restore reliability. This pattern highlights the influence of workload-specific mechanical factors on the observed changes in reliability, rather than contradicting the general trend of increased physiological noise at higher intensities.

To date, to the best of our knowledge, only our study and the study of Abt et al [4] used CPET during HR measurement in healthy volunteers. When comparing our findings with those reported by Abt et al, both studies demonstrate good agreement between smartwatch-derived and criterion HR values, although the magnitude of validity differs according to exercise intensity and device model. Abt et al, with 15 participants, found very high correlations (*r*=0.87‐0.98) and trivial bias for HRmax measurements obtained with the Apple Watch compared with a Polar reference, indicating strong validity at maximal effort. In contrast, our study, which included 55 participants, showed moderate to good reliability for the GW6 across exercise intensities, with the lowest agreement observed in the 80%‐90% HRmax range. Nevertheless, the MAPE remained small, supporting acceptable accuracy for practical use during exercise. Overall, the larger sample size of our study reinforces the consistency of these observations and suggests that, while optical sensors in current smartwatch models provide increasingly valid HR estimates, their performance may still vary across intensity domains and device types, particularly at submaximal workloads where motion artifacts are more likely.

In this study, the relative reliability (ICC) values obtained in the 5 HR zones are lower compared to those recently measured in individuals with coronary artery disease [5]. Kim et al [5] used an earlier-generation Samsung device (Galaxy Watch 4) and reported values above 0.90. The ICC values in this study ranged from moderate to excellent depending on exercise intensity, with the highest agreement observed at higher intensities (eg, 90‐100% HR zone), while lower-to-moderate ICCs were observed in the remaining zones. These discrepancies can be partially explained by differences in study designs. Kim et al [5] evaluated individuals with coronary artery disease and used the modified Bruce protocol, while in this study, we selected healthy individuals and used a running exercise test protocol with 1-minute stages with constant inclination. In addition, the higher ICC observed by Kim et al [5] may be due to participants being instructed to hold a handrail in front of them as a safety measure during the CPET. As the hand position was fixed during the test, generating less movement artifacts, HR accuracy may have been overestimated. Additionally, with the aim of determining the accuracy of wearable devices (among them, the Samsung Gear S) to measure HR at rest (in the supine and seated positions) and during exercise (walking and running on a treadmill and cycling on a cycle ergometer) in healthy individuals, Wallen et al [19] reported ICC values for HR of 0.80 (0.40 to 0.93). Our results are similar if we consider the 95% CIs.

The MAE measurement of the Samsung Galaxy Watch 4, as reported by Lima et al [20] under hands-free treadmill conditions (2.92 bpm), aligns closely with our findings, which demonstrated a range of 1.00 to 3.00 bpm, depending on the intensity zone. In addition, a coefficient of variation ≤10% was arbitrarily suggested as the standard criterion used to establish an acceptable level of reliability [21]. We consider acceptable accuracy values to be between 5%‐10%.

Aiming to investigate the HR measurement accuracy of 3 wrist-worn devices (Apple Watch 6, Polar Vantage V, and Fitbit Sense) during various activities (sitting, walking, running, resistance exercises, and cycling), Hajj-Boutros et al [22] showed that Apple Watch 6 appears to have the highest level of accuracy for HR monitoring for all 5 activities, followed by Polar Vantage V and Fitbit Sense. However, these investigations were conducted using task-based protocols with fixed intensities, which differ substantially from the physiological and biomechanical demands of a continuous incremental CPET. This study extends this body of evidence by evaluating HR accuracy across predefined intensity zones during a maximal ramp CPET, a context characterized by progressively increasing cardiovascular load, changing movement dynamics, and greater susceptibility to motion-related artifacts. This approach provides complementary and clinically relevant information that is not captured by activity-based protocols, particularly regarding device performance under conditions of rapid HR transitions and high exercise intensities.

In contrast to our findings, Thomson et al [3] reported that MAPE increased with exercise intensity during a Bruce protocol test, which consisted of 3-minute stages with progressively increasing speed and incline until voluntary fatigue. Heart rate reserve (HRR) was categorized into 5 intensity zones: very light (<20% HRR), light (20%‐40% HRR), moderate (40%‐60% HRR), vigorous (60%‐85% HRR), and very vigorous (>85% HRR). Their results showed MAPE increasing from ~2.4% at the lowest intensity to ~4.7% at the highest [3]. In this study, we found a reduction in MAPE with increasing intensity. These discrepancies can be partially explained by the differences in maximal tests (Bruce vs 1-min stages and constant incline), smartwatch models (Apple Watch vs Galaxy Watch), and handrail use during testing (hands-on vs hands-free), and possibly differences in skin color type, which could influence photoplethysmography readings. Although our subgroup analyses stratified by Fitzpatrick skin type did not reveal a consistent pattern of worsening performance across intensity zones, some differences in ICC estimates were observed between skin tone groups in specific zones. However, these differences should be interpreted with caution, given the limited sample size within subgroups.

Our findings suggest that the GW6 can provide reasonably reliable HR data during a maximal treadmill test, which may be of interest to clinicians, exercise-science practitioners, and active individuals who seek a convenient alternative to laboratory-grade equipment. The device should be regarded as a supplemental tool rather than a replacement for ECG-based measurements when precise rhythm analysis or diagnostic accuracy is required. In settings where cost or accessibility limits the use of more expensive monitors, GW6 offers a simple, affordable option for estimating HR, provided its limitations at near-maximal workloads are acknowledged. The use of stage-averaged HR in our study aligns with common practice in clinical exercise testing and CPET, facilitating physiological interpretation and comparability with previous studies. Although alternative approaches, such as shorter averaging windows or peak values within stages, may better capture rapid signal fluctuations, they may also be more susceptible to transient noise, particularly in wearable-derived measurements. Future studies specifically designed to compare different HR aggregation methods may help determine whether such alternatives provide additional value for device validation across different exercise intensities.

An operational aspect that emerged during data collection was the device failure rate of 22% (Figure 1), which reflects instances where the device did not generate usable HR data despite correct placement and adherence to protocol procedures. Although the focus of the study was accuracy during valid measurements, this level of attrition has practical implications. A failure rate of this magnitude may hinder consistent data acquisition in real-world laboratory or training environments, reducing the feasibility of using the device as a reliable continuous monitoring tool. This observation reinforces the need for device-specific robustness improvements and highlights the importance of considering both accuracy and operational reliability when evaluating wearable sensors.

### Limitations

Some limitations of this study should be highlighted. The devices were assessed in young, healthy participants exercising in a standardized laboratory setting. Therefore, our study can only be generalized for this population and setting only. GW6 was placed in a standard position, not considering the effect of left or right side on the accuracy of HR measurement. Therefore, future studies could consider testing watch side randomization to assess HR accuracy. HR measurement was performed using the Polar H10 chest strap, which has a good correlation with ECG. However, this should be considered a limitation.

Every test was stopped only after 2 objective or subjective signs of near-maximal effort were present. For the participants who terminated the test after satisfying the HR and RER criteria without reaching Borg>18, the “90%‐100% intensity zone” may correspond to a submaximal range (eg, ≈81%‐90% of true physiological HRmax). This could slightly overestimate device accuracy because the devices were not challenged at the absolute physiological limits of those individuals.

This study evaluated the validity of GW6 only during a maximal ramp test performed on a treadmill. The continuous, relatively rhythmic arm swing that occurs while running may differ substantially from the movement patterns seen in other common training modalities such as resistance training, high-intensity interval training, or cycling, where wrist motion can be more erratic, static, or intermittent. Therefore, the performance of the embedded photoplethysmography algorithm observed in this investigation may not be directly applicable to those activities. Readers are cautioned against generalizing these findings beyond treadmill-based exercise, and future work should examine accuracy across a broader range of exercise modalities.

The study encountered a 22% device failure rate, leading to the exclusion of 17 participants due to unusable HR data. While the primary aim was to investigate accuracy during valid recordings, this attrition rate represents a limitation of the operational performance of the device. Such failures may impact the practical use in laboratory or field settings, and future research should investigate underlying causes and potential technical improvements to enhance data reliability. It should be noted that if device failure is correlated with specific physiological characteristics (eg, darker skin tone or specific arm movements), the remaining valid sample (n=55) may represent a “best-case” scenario, potentially inflating the accuracy estimates relative to the general population. However, the subgroup analyses stratified by skin tone according to the Fitzpatrick classification did not demonstrate statistically or clinically meaningful differences in device performance.

Additionally, we analyzed a potential survivability bias to identify any patterns among participants excluded. Initially, we conducted an empirical analysis of demographic variables between the failure group and the valid group, focusing on BMI, age, and skin pigmentation. No significant patterns were found in these variables. A second analysis was conducted to address concerns about failed trials from female participants, under the hypothesis that smaller wrist sizes might have affected the proper accommodation of smartwatches. However, this analysis also revealed no discernible patterns. While no bias was identified in the initial analysis, further investigations may be necessary.

### Conclusions

This study indicates that the GW6 photoplethysmography-based technology demonstrated reasonable validity in monitoring HR during a maximal cardiopulmonary exercise test in healthy participants.

## Abbreviations

CPET: cardiopulmonary exercise testing
CV: coefficient of variation
ECG: electrocardiogram
GW6: Galaxy Watch 6
HR: heart rate
HRR: heart rate reserve
ICC: intraclass correlation coefficient
LoA: limit of agreement
MAE: median absolute error
MAPE: median absolute percentage error
RER: respiratory exchange ratio
RMSE: root mean square error
STROBE: Strengthening the Reporting of Observational Studies in Epidemiology
